# Case report: Severe dry cough associated with superior vena cava syndrome—Caused recurrent chylothorax

**DOI:** 10.3389/fcvm.2022.970783

**Published:** 2022-11-17

**Authors:** Ziming Wan

**Affiliations:** Department of Nephrology, The First Affiliated Hospital of Chongqing Medical University, Chongqing, China

**Keywords:** chylothorax, dry cough, enhanced computed tomography, superior vena cava syndrome, case report

## Abstract

**Introduction:**

Symptomatic pleural effusion is occasionally caused by superior vena cava syndrome. Dyspnea and pleuritic chest pain are common symptoms of pleural effusion. However, the current literature has not reported a causal linkage between chylous pleural effusion and dry cough.

**Case presentation:**

A patient with uremia suffered from an unexplained severe dry cough, which could be triggered by postural changes. Medical examinations ruled out the possibility of chronic bronchitis, gastroesophageal reflux, chest tumor, tuberculosis, asthma, chronic obstructive pulmonary disease, and allergy history. Examinations showed that the patient had chylous pleural effusion. The cough symptoms were relieved after extraction of the pleural effusion but soon reappeared with the recurrence of chylothorax. Enhanced computed tomography showed that the patient had superior vena cava occlusion. After recanalization of the superior vena cava by percutaneous balloon dilatation, the patient no longer had chylothorax, and the severe cough was eliminated.

**Conclusion:**

Super vena cava syndrome can cause chylothorax and further stimulate severe dry cough. Cough is not a specific symptom. Chest imaging and pleural fluid analysis can help narrow down the diagnosis.

## Introduction

Pleural effusion refers to the fluid accumulated in the pleural cavity and is usually divided into transudate and exudate. Thoracentesis and pleural effusion analysis are often performed to determine the cause. Chylous pleural effusion, or chylothorax, is a non-inflammatory exudate rich in triglycerides (1). Chylothorax is usually caused by the destruction of the thoracic duct due to trauma, thoracic tumors, severe infection, and occasionally by superior vena cava syndrome. In cases of chylous collection of > 500 mL, shortness of breath, chest tightness, palpitation, and dyspnea may occur (2).

Patients with chronic coughs are very common in the respiratory department outpatient clinic, but they are often ignored by clinicians. Many patients are generally diagnosed with bronchitis or chronic bronchitis, which is ineffective after cough and expectorant treatment or a variety of antibiotics. The diagnosis of a chronic cough generally follows the sequence of simple examination before complex examination and common diseases before rare diseases. Chronic cough is not a specific symptom. The most frequent causes include eosinophilic bronchitis, postnasal drip syndrome, gastroesophageal reflux disease, cough variant asthma, and upper airway cough syndrome. Cough receptors are found not only in the throat, trachea, bronchus, and other parts of the respiratory system, but also in the esophagus, paranasal sinuses, external auditory canal, pleura, pericardium, and other parts (3). Therefore, diseases of the above systems or parts may produce cough symptoms. The key to chronic cough treatment lies in determining the etiology.

No previous studies have reported a causal and therapeutic linkage between large chylous pleural effusion and cough. This article reports a severe dry cough case caused by superior vena cave occlusion-induced recurrent chylothorax.

## Case report

**FIGURE 1:**
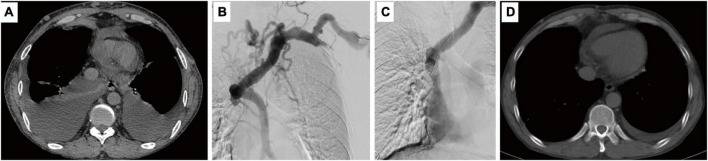
Computed tomography shows pleural effusion **(A)** and angiography shows superior vena cava occlusion **(B)**. Balloon dilatation was performed **(C)** and pleural effusion no longer appeared after 4 months **(D)**.

A 50-year-old man sought treatment in several hospitals because of a severe dry cough. The cough first appeared in December 2020 without obvious inducement, accompanied by a small amount of white foam sputum and sticky sputum. During 1 month, the patient’s cough gradually worsened, with dyspnea after activities and paroxysmal dyspnea at night. Cough symptoms were associated with postural changes, such as standing up from sitting, bending over, or turning over during sleep, which could trigger severe coughs, and the patient even had transient amaurosis (self-reported syncope three times). The patient was hospitalized at Xinqiao Hospital of Army Military Medical University (Chongqing, China) on 11 January 2021, and discharged on 21 January 2021. Computed tomography (CT) of the chest showed bilateral pleural effusion (Figure 1A), which was later tested to be chylous fluid (Table 1). The cough was relieved after extracting the pleural fluid. However, the relief could only last for 1 day until the pleural effusion reappeared. The patient had to have pleural effusion extracted every other day to relieve his cough, and the amount of pleural effusion was very large (800–1,000 mL each time). It was suspected that the severe cough was related to recurrent pleural effusion. To determine the cause of such a serious pleural effusion, the patient received enhanced CT, which found an occlusion in the superior vena cava. Because the patient had a long history of dialysis because of uremia, he was recommended to go to the nephrology department to treat his superior vena cava occlusion.

**TABLE 1:** Results of pleural effusion analysis.

	Jan 21, 2021	Feb 21, 2021	Feb 23, 2021
**Color**	Right: clear yellow Left: milky	Yellowish red and cloudy	Yellowish white and cloudy
**Rivalta test**	+	+	2+
**Chyle test**	+	+	+
**Total cells (/L)**	N/A	3.61 × 10^9^	1.33 × 10^9^
**Nucleated cells (/L)**	N/A	6.1 × 10^8^	3.3 × 10^8^
**Lymphocytes**	N/A	90%	96%
**Total protein (g/L)**	29.5	N/A	26
**Albumin (g/L)**	N/A	13	13
**Lactic dehydrogenase (U/L)**	129.5	89	88
**Adenosine deaminase (U/L)**	4	1.4	4.6

The patient then visited the Department of Nephrology of our hospital. The patient was diagnosed with chronic kidney disease stage 5 in 2003 and received kidney transplantations in 2003 and 2017. The second transplantation failed. He had undergone dialysis for 8 years. In the first 6 months, a right internal jugular vein long-term catheter was used for dialysis, which was later changed to a left brachiocephalic arteriovenous fistula. Physical examination showed that the patient had erythema and edema on the face and neck, diffuse varicosities across the anterior chest, and distended jugular veins. Subsequent CT venography and digital subtraction angiography showed occlusion of the superior vena cava (Figure 1B) and extensive venous collateral vessels within the lower neck and superior mediastinum. The patient was admitted for hospitalization on 20 February 2021. To determine the cause of pleural effusion, 40 mL of pleural fluid was drawn from the right thoracic drainage tube and sent for pleural effusion analysis, exfoliated cell examination (thoracic tumor), and bacterial culture (tuberculosis infection).

The results of the pleural effusion analysis returned on 21st February, showing chyle positivity with elevated lymphocytes (Table 1). No abnormality was found in exfoliated cell examination. Ward rounds showed that the patient’s temperature was normal, superficial lymph nodes were not palpable, the breath sounds were low and clear, and no rales were heard. Routine blood tests showed that leukocytes (7.77 × 10^9^/L) and neutrophils (86.5%) were elevated. Because the inflammatory indicators increased in the pleural effusion and blood samples, the patient was given piperacillin-tazobactam (4.5 g q12 h) as treatment. At 1:00 p.m. that day, the patient experienced transient syncope. Sudden loss of consciousness occurred after coughing induced by positional changes from lying to sitting. The patient fell to the ground, without limb twitching, incontinence, or foaming at the mouth. The patient improved spontaneously after 5 s and did not complain of dizziness, headache, palpitations, chest tightness, or dyspnea. His blood pressure was 105/63 mmHg, and his heart rate was 88 times/min. No special treatment was carried out for this syncope.

On 22 February, several tests, including the detection of anti-tuberculosis antibody, sputum smear, sputum culture, and detection of acid-fast bacilli in sputum, were performed. The patient had been taking immunosuppressants since renal transplantation, which might increase the susceptibility to tuberculosis infection. Tuberculosis infection is a possible cause of chylothorax, so we decided to further search for evidence of tuberculosis infection. The bacterial culture of the pleural effusion showed positive results for *Staphylococcus lugdunensis*, so the antibiotic treatment was continued.

On 23 February, 1,000 mL of effusion was extracted from both lungs through thoracentesis. The pleural fluid analysis showed that the total cell counts decreased, suggesting that the antibiotic treatment was effective. Antibody tests for tuberculosis (gold labeled immunoassay) were weakly positive (+/-), but no *Mycobacterium tuberculosis* was detected in sputum specimens (sandwich cup method). According to the current examination results, the possibility of tuberculosis infection could be ruled out. Therefore, we speculated that superior vena cava occlusion was the most likely cause of recurrent chylothorax.

On 24th February, percutaneous balloon dilatation was performed to recanalize the superior vena cava (Figure 1C). The superior vena cava was clearly visualized and unobstructed. The collateral circulation mostly disappeared, and the stenosis was relieved.

The patient recovered well in the following days. Physical examination showed that the swelling of the face and neck obviously subsided. Three days after the operation (March 1), the patient received an ultrasound examination for the detection of pleural effusion. The results showed a flaky anechoic area in the bilateral chest, with a maximum depth of 46 mm in the right chest and 40 mm in the left chest. The location of the pleural fluid was not determined due to the small amount. On 3rd March, the patient received a chest CT again. Only a small amount of pleural effusion was found, and most of the lungs were dilated. The patient was discharged on the same day.

Follow-up was conducted after 4 months and 12 months. Chest CT showed no pleural effusion (Figure 1D). The patient reported that cough symptoms were eliminated. Based on the results, we can confirm that the obstruction of the superior vena cava caused the patient to develop recurrent chylothorax, and further stimulated severe postural change-related cough.

## Discussion

In general, symptomatic pleural effusion mainly causes dyspnea or pleuritic chest pain. The rarity of the present case is that a severe chronic cough related to postural changes is the main symptom. In this case, the patient’s complex complications increase the difficulty of diagnosis. The patient had received kidney transplantation twice because of uremia and had complications, including renal hypertension, renal hypoproteinemia, hyperlipidemia, and pericardial effusion. Chest imaging examination is helpful to narrow down the diagnosis. Fortunately for this case, thoracentesis, and pleural effusion analysis were performed immediately after the discovery of pleural effusion, which suggested that the cough symptoms were caused by chylothorax. We found another rare case report of chronic cough and recurrent pleural effusion (4). Similarly, in that case, the doctors performed chest radiography after excluding other common causes of cough and found pleural effusion. The pleural effusion was exudate. After consecutive examinations, the patient was diagnosed with constrictive pericarditis. These two cases illustrate that the diagnosis of unexplained cough can be very challenging.

Although it took several days to determine the cause of chylothorax, the exploration direction in the diagnosis process was clear. The three most likely causes were thoracic tumor, tuberculosis infection, and superior vena cava syndrome. Thoracic tumors were first excluded by enhanced CT and exfoliated cell examination of the pleural effusion. Infection also did not seem to be the cause because a large amount of pleural effusion is often related to a very serious infection. The current case only showed a slight increase in inflammatory indicators, and his body temperature remained normal. Combined with a bacterial culture of pleural effusion, tuberculosis antibody screening, and tuberculosis detection in sputum specimens, the presence of tuberculosis was ruled out. Considering the patient’s dialysis history, the obvious physical appearance of central vein occlusion, and the angiography evidence, superior vena cava syndrome was the most likely cause of chylothorax. Finally, the recanalization operation eliminated the symptoms of chylothorax and cough at the same time, which confirmed our diagnosis.

In summary, super vena cava syndrome can cause chylothorax and further stimulate severe dry cough. Chest imaging and pleural fluid analysis can help narrow down the diagnosis of cough.

## Patient perspective

The ultimate cause of the patient’s cough was superior vena cava obstruction, which is a common complication in dialysis patients. However, no nephrologist had established a correlation between dialysis and cough during the patient’s routine dialysis. The reason is that pleural effusion caused by the superior vena cava is not common, and cough caused by pleural effusion is extremely rare. Patients with cough symptoms usually go to the respiratory department for diagnosis and treatment. In this case, the patient had visited several hospitals before finally being diagnosed in our hospital and had been unable to receive effective treatment. The patient’s quality of life quality was seriously affected, and the patient showed obvious anxiety and disappointment. The patient was finally diagnosed and effectively treated in our hospital. On the second day after the operation, the patient reported that his cough and dyspnea after activities had improved significantly. During the follow-up, both large pleural effusion and cough no longer recur. The patient and his family were very satisfied and expressed thanks to the relevant medical staff.

## Data availability statement

The original contributions presented in this study are included in the article/supplementary material, further inquiries can be directed to the corresponding author.

## Ethics statement

Ethical review and approval was not required for the study on human participants in accordance with the local legislation and institutional requirements. The patients/participants provided their written informed consent to participate in this study. Written informed consent was obtained from the individual(s) for the publication of any potentially identifiable images or data included in this article.

## Author contributions

ZW was the responsible physician in the treatment process of the patient and collected all the data, wrote the article, and approved it for publication.
